# Dynamically monitoring lymphatic and vascular systems in physiological and pathological conditions of a swine model via a portable NIR-II imaging system with ICG

**DOI:** 10.7150/ijms.71956

**Published:** 2022-10-17

**Authors:** Zheng Wang, Yifeng Yu, Yifan Wu, Siqi Gao, Lanping Hu, Chao Jian, Baiwen Qi, Aixi Yu

**Affiliations:** Department of Orthopedics Trauma and Microsurgery, Zhongnan Hospital of Wuhan University, Wuhan, Hubei 430071, P. R. China.

**Keywords:** NIR-II imaging, surgical navigation, microsurgery, diagnosis, vascular system malfunction, lymphatic system malfunction

## Abstract

**Objective:** NIR-II imaging with indocyanine green (ICG) has been clinically used in liver tumor resection. However, few data are available concerning the application of ICG-NIR-II in lymphatic and vascular systems in clinic. To expand the application and promote the clinical translation of this approach, we aimed to investigate the feasibility of ICG-NIR-II imaging for monitoring both lymphatic and vascular systems in physiological and pathological conditions using a swine model and compared it to ICG-NIR-I imaging.

**Methods:** we constructed a portable NIR-II imaging system suitable for large animals. Different simulated clinical scenarios in lymphatic and vascular systems of pigs, including lymphatic drainage, lymphorrhea, lymphatic obstruction, lymphatic reconstruction in flaps, venous thrombus formation and vascular anastomosis were modeled to evaluate the reliability of our NIR-II imaging system and the imaging quality of ICG in the NIR-I/II window.

**Results:** Under different simulated clinical scenarios, our portable NIR-II imaging system showed good reliability for pigs. With the help of the portable imaging system, dynamical visualization of lymph vessels, lymph nodes and blood vessels of pigs in different clinical scenarios could be achieved in NIR-II imaging by using the tail fluorescence of ICG. Moreover, ICG-NIR-II imaging has lower background fluorescence and higher resolution than ICG-NIR-I imaging.

**Conclusions:** We demonstrated the first application of a portable NIR-II imaging system for dynamically monitoring both lymphatic and vascular systems in physiological and pathological conditions using a swine model. Our study indicates that ICG-NIR-II imaging be a promising approach for the diagnosis of malfunctions in lymphatic and vascular systems and the surgical navigation of microsurgery and reconstructive surgery.

## Introduction

Diseases of the vascular and lymphatic systems, including vascular and lymphatic system malfunctions, such as lymphorrhea, lymphedema and thrombus formation, are quite common and pose a severe threat to human health 1-4. Therefore, real-time visualization for physiological or pathological processes of vascular and lymphatic systems *in vivo* is an essential goal in both research and clinic. But conventional techniques including X-ray, ultrasonography (US), magnetic resonance imaging (MRI) and computed tomography (CT) cannot reach our requirement, since that the vasculature is a closed system 5, 6. As a radiation-free imaging modality with high resolution and sensitivity, fluorescence-based near-infrared (NIR) imaging has been a hotspot in real-time visualization and monitoring dynamic biological processes in living subjects 7-9.

Previously, commercial fluorescence imaging systems with silicon detectors were only sensitive at wavelengths between 700-900 nm, hampering deeper tissue imaging. Currently, improvements in indium gallium arsenide (InGaAs) cameras capable of detecting the spectra in the NIR-II window (1000-1700 nm) have significantly propelled the development of NIR-II detection technology 10. Fluorescence imaging in the NIR-II window holds considerable prospect for *in vivo* imaging to obtain detailed information of multiple physiological and pathological processes including vascular and lymphatic systems, due to reduced scattering and low auto-fluorescence of normal tissue, higher spatial resolution and deeper tissue penetration when compared to imaging in the NIR-I window (650-900 nm) 11, 12. As a result, NIR-II imaging has attracted more attention recently and been considered to exhibit great prospects for clinical translation 5, 6.

Despite many advantages and extensive validation in animal models, the application of NIR-II imaging for patients is still impeded by a lack of limited availability of suitable optical probes 13. Even though various NIR-II fluorophores, including organic polymers and inorganic nanoparticles have been designed and synthesized 14-18, there is still a lack of evaluation of their efficacy and safety in clinical settings. Consequently, it is of great significance to find a reliable FDA-approved NIR-II agent to accelerate the use of NIR-II imaging in clinic. Indocyanine green (ICG) is a traditional NIR-I fluorescent agent approved by the FDA in 1950s and has been widely used in angiography 19, hepatic clearance test 20, laparoscopic surgery 21, sentinel lymph node mapping 22 and tumor resection guidance 23. Recently, it has been demonstrated that this conventional NIR-I dye, when excited with a 780 nm laser source, was also capable of fluorescing in the NIR-II window that can be detected by an InGaAs camera, known as tail fluorescence 24-33. Thanks to the discovery, ICG-NIR-II imaging has been clinically used in liver tumor resection and proved to be superior to traditional ICG-NIR-I imaging 34. However, unlike the enhanced permeability and retention (EPR) effect of ICG in tumors, ICG would be quickly cleared by healthy liver after entering the vasculature. Therefore, some differences may exist between the imaging of circulatory malfunctions and tumors by ICG-NIR-II imaging.

To date, few data are available concerning the clinical application of ICG-NIR-II imaging in the circulatory system. Due to technical limitations, current devices for NIR-II imaging are integrated, complex and cannot be moved around, and can only be used in the laboratory, resulting that this approach has been reported to be limited to small blood vessels of rodents in most preclinical studies 26-28. Even though there have been several studies of cerebral vascular NIR-II imaging with ICG in pigs and macaques, the devices used in these experiments were not portable 31-33. In addition, the observations have been reported exclusively in vascular system, and ICG-NIR-II imaging in lymphatic system has not been reported. To promote the clinical translation of this approach in the diagnose of malfunctions in both lymphatic and vascular systems, more suitable conditions need to be explored in animal models which more closely represent humans in size and complexity.

In this preclinical study, we constructed a portable NIR-II imaging system suitable for large animals and investigated the effect of ICG-NIR-II imaging for lymphatic and vascular systems in physiological and pathological scenarios using a swine model. We also compared this approach to the conventional ICG-NIR-I imaging, to provide additional context to the results.

## Materials and Methods

### Animals

All experiments were carried out under the Guide for the Care and Use of Laboratory Animals prepared by the National Institutes of Health and approved by the Experimental Animal Welfare Ethics Committee of Zhongnan Hospital of Wuhan University under animal protocol number ZN2021070. Eighteen farm pigs, weighing 20-25 kg, and purchased from the Experimental Animal Center of Wuhan University (Wuhan, China) were used for this research. Premedication by the intramuscular injection of ketamine (20 mg/kg) was administered 10 min before surgery. Anesthesia was induced with intravenous propofol (4 mg/kg) followed by maintenance with continuous pumping of propofol (0.3mg/kg/h).

### NIR-I/II imaging instrument

We constructed a portable NIR-II imaging system suitable for pigs, covering the spectrum range of 900-1700 nm (Figure S1). The system is composed of an ultra-low temperature air cooling In GaAs camera (640 × 512 pixels, Hongbin, China) equipped with a prime lens (focal length: 50 mm, antireflection coating at 0.8-1.8μm, Edmund Optics), cooled to -80 °C. The long-pass filters (ThorLabs, USA) vary from 900-, 1000-, 1100-, 1200-, and 1300-nm, were utilized to extract different NIR-II fluorescence signals as required.

Images in the NIR-I window were captured using a silicon camera (1920 × 1080 pixels, ThorLabs, USA) equipped with a lens (focal length: 35 mm, ThorLabs, USA), which was fitted with an 800-nm long-pass filter and a 900-nm short-pass filter to filter away 785 nm excitation and extract NIR-I fluorescence signal, respectively.

A 785-nm laser device (Lasever, China) was used to provide uniform illumination on the field for NIR-I and NIR-II imaging synchronously. The facular power density was adjusted to 10-15 mW/cm^2^. Animals were positioned in a supine position, and the NIR-II imaging system was fixed in place as required. The NIR-I imaging system was placed in parallel with the NIR-II imaging system.

### NIR-II imaging for preoperative mapping of lymphatic channels and sentinel lymph nodes

A total of 1 ml ICG (2.5 mg/ml, Dandong Pharmaceutical Factory, China) was injected intradermally into the footpad of the pig's left hind leg (n=3). To assess the most suitable filter, the fluorescence images of the lymphatic drainage were captured in the NIR-II window sequentially using 900-, 1000-, 1100-, 1200-, or 1300-nm long-pass filters 5 minutes after injection. Then, images in NIR-I window were collected and compared to those in NIR-II window to evaluate the imaging quality of NIR-I/II imaging.

Subsequently, the inguinal lymph node and one of the lymph vessels were marked on the surface of skin with a felt-tipped pen under the fluorescent imaging. Directly intraoperative localization using methylene blue staining was applied as an objective control. A total of 1 ml methylene blue (10 mg/ml, Jicuan Pharmaceutical Factory, China) was injected at the same sites as the ICG injections. Subsequently, a 4 cm incision was made on the surface projection to reveal lymph vessels and lymph nodes stained with methylene blue in the superficial fascia layer. The inguinal lymph node and a short segment of lymph vessel were harvested and analyzed with H&E stain.

### NIR-II imaging for lymphorrhea and lymphatic obstruction

Lymphorrhea and lymphatic obstruction models were established by ligating and transecting lymph vessels of pigs, respectively (n=3). In brief, 1ml methylene blue was injected intradermally into the footpad, a lymph vessel was identified in the superficial fascia layer of the left hind leg. Then, the lymph vessel was transected for the lymphorrhea model and ligated by using 7-0 nylon sutures for the lymphatic obstruction model. The incision was closed in layers. Subsequently, 1 ml ICG was injected intradermally into the left footpad. Five minutes later, the affected limb was under NIR-II camera to detect the ligation and leakage site.

### NIR-II imaging for monitoring lymphatic reconstruction in the saphenous artery flap

**Figure 1:**
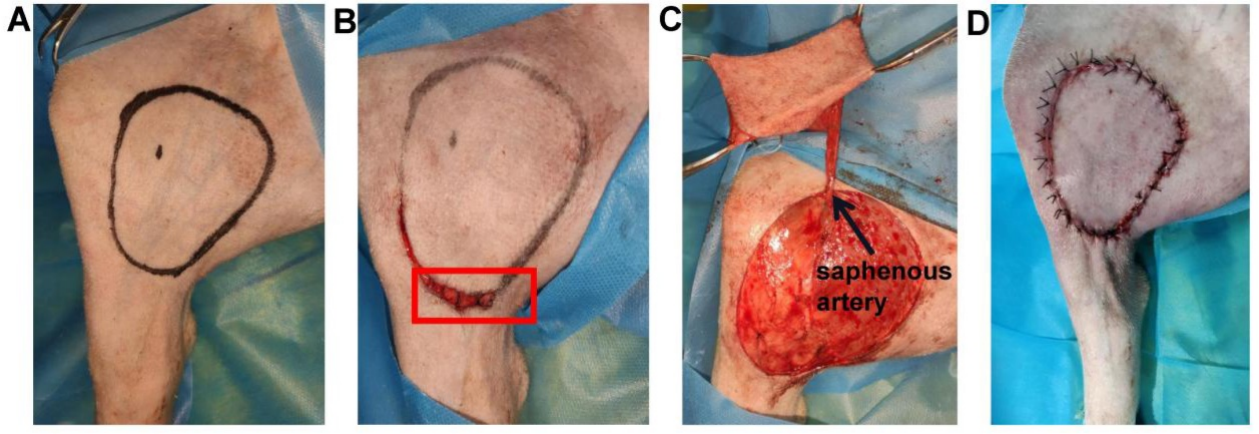
** The saphenous artery flap of the pig was harvested. (A)** An ellipse containing all the lymphatic vessels stained with methylene blue was drawn on the area below the groin. **(B)** The broken lymph vessels stained with methylene blue outside the flap were ligated (red box). **(C)** The flap was peeled, and the proximal saphenous artery served as the axial vessel of the flap. **(D)** The flap was sutured in situ.

The saphenous artery flap of the pig was harvested in a supine position (n=3) (Figure 1A). Five minutes before the operation, 1 ml injection of methylene blue was performed intradermally into the footpad of the pig's left hind legs. Soon afterwards, superficial lymph vessels, running parallel to the saphenous vessels were visualized. An ellipse of the flap outline containing all the lymphatic vessels stained with methylene blue was drawn with a surgical marker on the area below the groin, according to the surface projection of the saphenous artery in pigs 35. The skin incision was firstly performed at the proximal and distal parts of the ellipse to look for and transect subdermal lymph vessels stained with methylene blue. To prevent lymphatic leakage, all the broken lymph vessels stained with methylene blue outside the flap were ligated by using 7-0 nylon sutures (Figure 1B). Subsequently, the skin flap was peeled from the vastus medialis and gracilis searching for the saphenous artery. The saphenous artery was transected and ligated distally, and the proximal saphenous artery served as the axial vessel of the flap (Figure 1C). The flap was then sutured in situ (Figure 1D). NIR-I/II images after intradermal injection of 2ml were captured at different time points (0, 7, and 21 days) post-operation to monitor the reconstruction of the lymphatic system in the flap.

### NIR-II imaging for normal femoral vessel system

Under sterile conditions, a 10 cm longitudinal incision was made below the inguinal ligament. By dissecting the fascia, exposing and preparing the sartorius muscle, the right femoral artery and vein were carefully isolated about 5 cm long segment above the level of the great saphenous vein entering the femoral vein. Subsequently, 2 mL ICG was injected through the pig's auricular vein. Images were captured at different time points (30 s, 2 min, 5 min, and 8 min) post-injection (n=3).

### NIR-II imaging for femoral venous thrombus formation

To induce femoral venous thrombus, the vascular injury was generated by wrapping with a 0.5 × 1 cm swatch of grade 3M Whatman filter paper saturated with a 50% solution of FeCl_3_ 36 for 30 minutes. Then, 2 mL ICG was injected through the pig's auricular vein, and NIR images were recorded immediately after injection to visualize the thrombus location (n=3).

### NIR-II imaging for vascular patency detection during anastomosis

The right femoral artery was carefully exposed about 5 cm above the level of the great saphenous vein entering the femoral vein in each animal. Then, two vascular clamps were placed in the distal and proximal parts of the exposed artery, and the femoral artery was transected at the central part. The anastomosis was performed in end-to-end fashion by using 7-0 nylon sutures. After accomplishing the anastomosis, 2 mL ICG was administered intravenously and NIR-I/II imaging were performed to assess the arterial patency (n=3). Finally, direct examination using the vascular patency test was applied as an objective control.

### Statistical analysis

Quantitative analysis of each NIR fluorescence signal intensity was performed through the Image J software (National Institutes of Health, Bethesda, MD). Quantitative results are expressed as mean ± standard deviation (SD), and GraphPad Prism7 (GraphPad Software, Inc.) was used for data analysis. Student's t-test was employed to compare the quantitative data between the NIR-II and NIR-I imaging. Statistical significance was assigned for *P* value < 0.05.

## Results

### NIR-II imaging for preoperative mapping of lymphatic channels and sentinel lymph nodes

**Figure 2:**
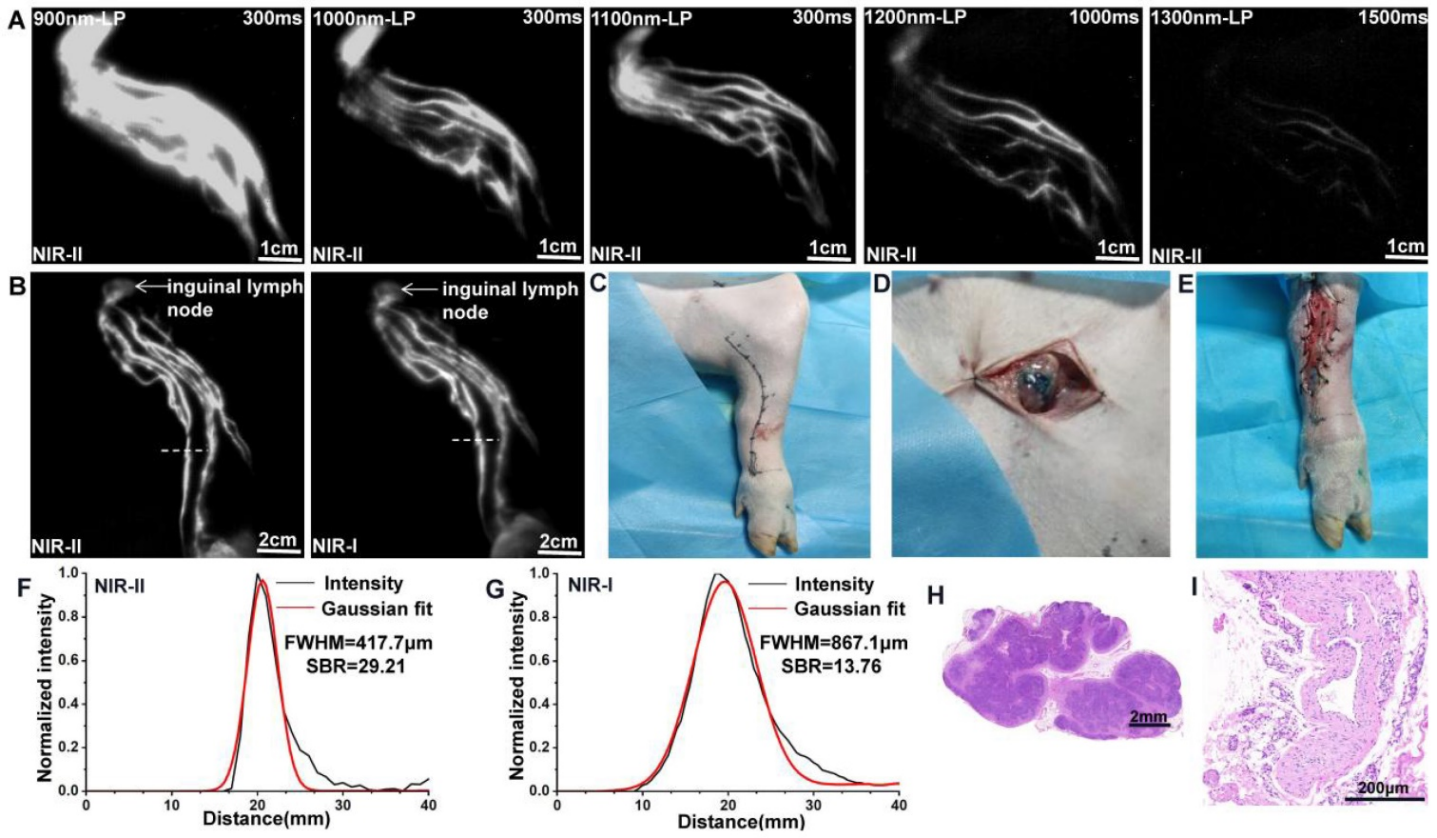
** NIR-II imaging for preoperative mapping of lymphatic channels and lymph nodes. (A)** The lymphatic drainage of the pig's left hind leg was imaged in NIR-II window with 900-, 1000-, 1100-, 1200-, and 1300-nm long-pass filters. **(B)** Fluorescence images of the lymphatic drainage in NIR-II/I window, respectively. **(C)** Preoperative localization of the inguinal lymph node (black dot) and a lymph vessel (black curve). **(D and E)** The inguinal lymph node and lymph vessel (stained with methylene blue) were found in the marked subcutaneous area. **(F and G)** The FWHM and SBRs of lymph vessels (white dash lines in Figure B) were calculated, respectively. **(H and I)** The dissected lymph node and lymphatic vessel was confirmed by H&E stain.

After intradermal injection of ICG, the background signal around the lymphatic channels and sentinel lymph nodes (SLNs) decreased significantly when imaging was performed in more extended wavelength regions (Figure 2A). However, the fluorescence intensity concomitantly decreased, and a longer exposure time was needed for imaging with filters above 1200-nm, because more fluorescence was filtered out. The results suggested that a 1100-nm filter enabled a better contrast and a higher resolution for our NIR-II imaging system. Consequently, an exposure time of 300 ms and a 1100-nm filter were set for NIR-II lymphatic system imaging. The lymph vessels connecting the injection site with the inguinal lymph node were notably distinguished, providing a comprehensive understanding of the real-time lymphatic drainage in living subjects (Figure 2B) and enabling us to achieve precise location of the inguinal lymph node (marked by a black dot) and the lymphatic vessel (marked by a black curve) preoperatively (Figure 2C). Movie S1 shows this in more detail. According to the fluorescence, the lymph vessel and inguinal lymph node were also found in the marked subcutaneous area during operation (Figure 2D and E), corresponding to methylene blue staining.

We also compared the resolution of lymph vessel and lymph node imaging in NIR-I and NIR-II window using the Gaussian-fitted full-width-half-maximum (FWHM), based on the cross-sectional intensity profiles. Compared to NIR-I imaging, the lymph vessel (marked by a white dash line in Figure 2B) visualized in the NIR-II window demonstrated significant enhanced feature sharpness (417.7 μm vs 867.1 μm; Figure 2F and G). The SBR of lymph vessel (defined as the lymphatic vessel-signal-to-skin ratio) obtained from NIR-II imaging was 29.21, which was higher than the corresponding NIR-I imaging 13.76. Statistical analyses revealed higher SBR (Figure S2A, P < 0.001) and lower FWHM (Figure S2B, P < 0.001) in the NIR-II window than those in the NIR-I window. The dissected lymph nodes and lymph vessels were confirmed by H&E stain (Figure 2H and I).

### NIR-II imaging for the lymphorrhea and lymphatic obstruction

**Figure 3:**
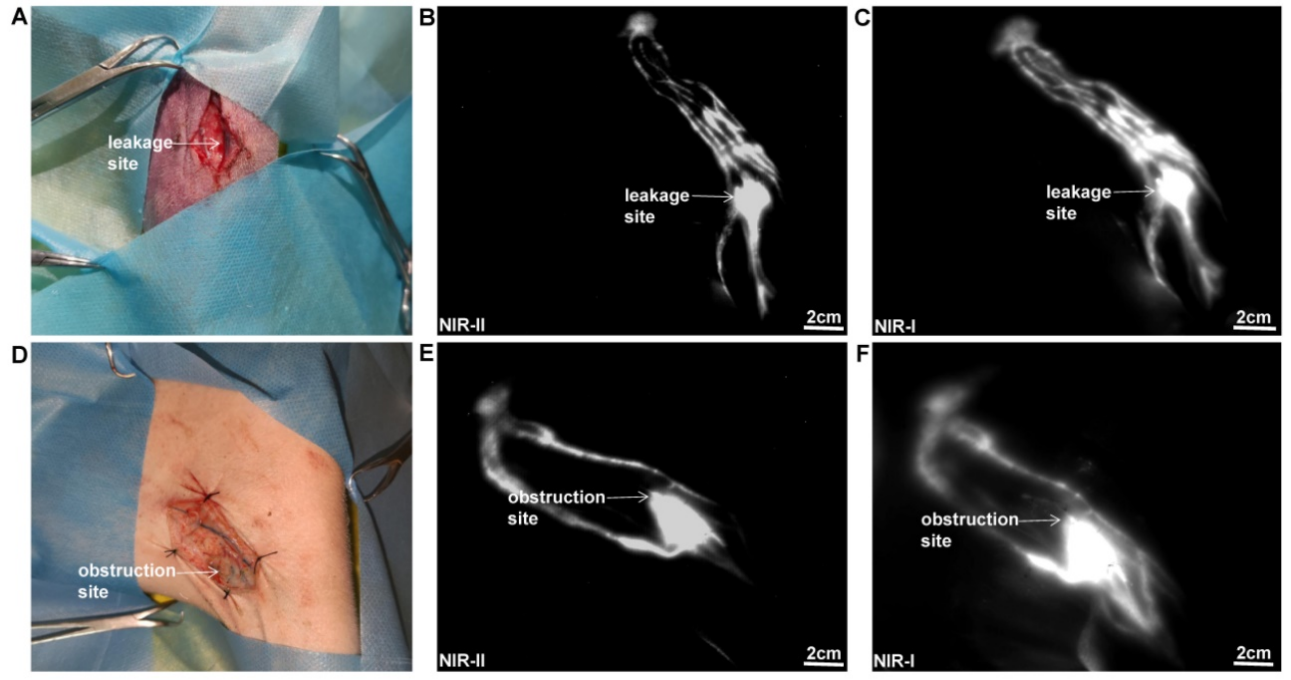
** NIR imaging for the lymphorrhea and lymphatic obstruction. (A-C)** Bright-field, NIR-II window, and NIR-I window images of lymphorrhea injury. **(D-F)** Bright-field, NIR-II window, and NIR-I window images of lymphatic vessel ligation injury.

As displayed in Figure 3A, a lymphatic vessel transection injury model was established. The specific leakage site could be well identified in NIR-II window (Figure 3B), with a reduced background signal compared to that in NIR-I window (Figure 3C). As for the lymphatic obstruction model (Figure 3D), the ligation site and dilated distal lymphatic vessel were easily identified in the NIR-II window (Figure 3E), outperforming the imaging in the NIR-I window (Figure 3F).

### NIR-II imaging for monitoring lymphatic reconstruction in the saphenous artery flap

**Figure 4:**
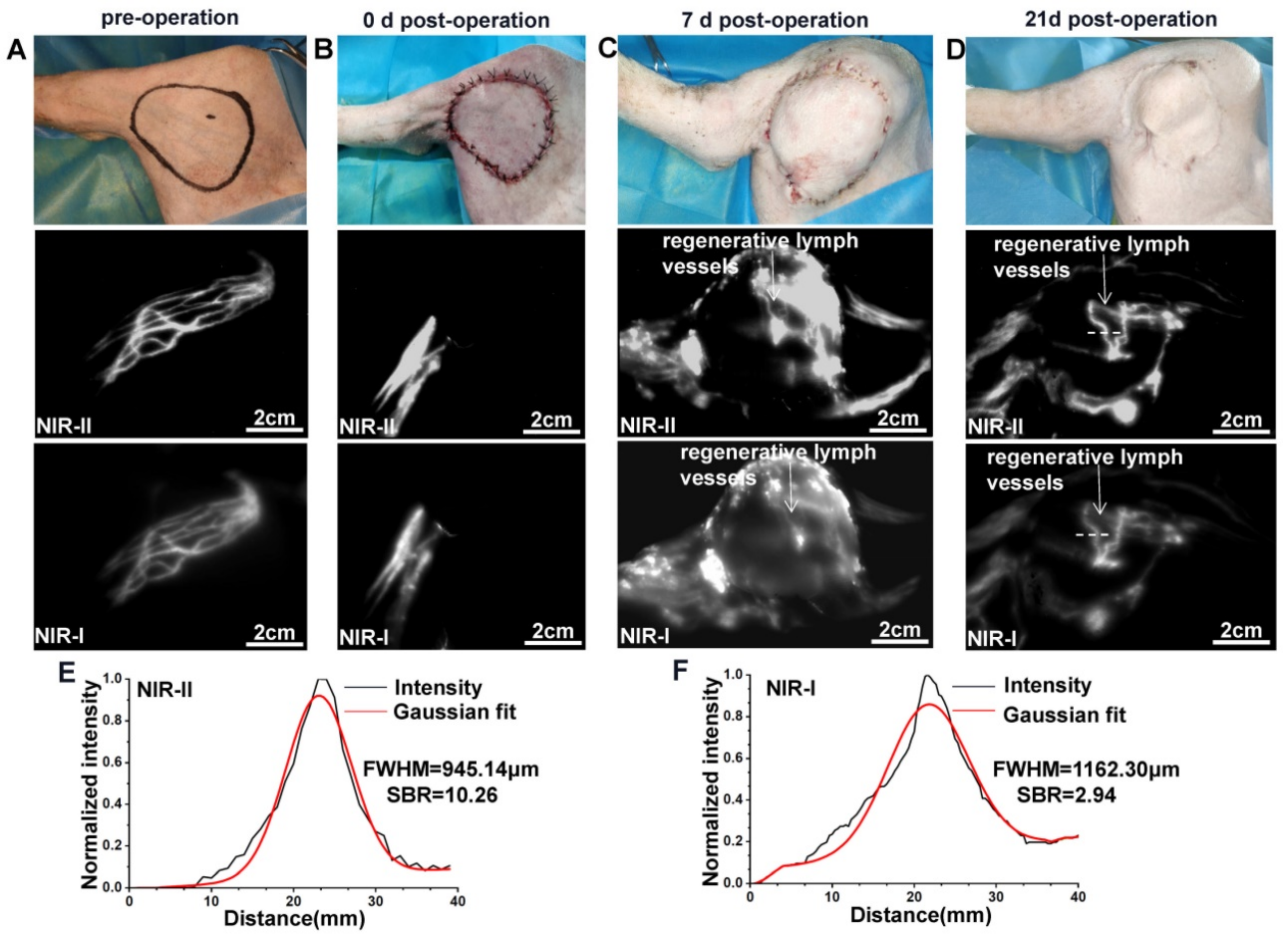
** NIR imaging for monitoring lymphatic reconstruction in the saphenous artery flap. (A-D)** Bright-field, NIR-II window and NIR-I window images of the surgical area before operation and 0, 7 and 21 day after flap resection, respectively. **(E and F)** The FWHM and SBRs of regenerative lymph vessel (white dash lines in Figure **D**) were calculated, respectively.

Normal lymphatic drainage was shown in the surgical area before the operation (Figure 4A), while no fluorescence could be seen in the flap after intradermal administration of ICG on day 0 post-operation (Figure 4B). We further dynamically traced the same model on day 7 and found that the whole flap was edematous evidently (Figure 4C). Meanwhile, the ICG fluorescence showed only a small number of new lymph vessels with obvious splash, star cluster or dispersion shape in the proximal part of the flap, and the lymphatic drainage reconstructed following the direction to the groin lymph node. On day 21, the edema at the proximal part of the flap had almost disappeared, while edema at the distal part could still be observed obviously (Figure 4D). Correspondingly, ICG fluorescence showed more lymphangiogenesis in the proximal part of the flap, but few lymphangiogenesis in the distal part. Movie S2 shows this in more detail.

The FWHM of the regenerated lymph vessels within the flap marked by white dash lines in Figure 4D were calculated as 945.14 μm and 1162.30 μm, respectively. The SBR was calculated as 10.26 in NIR-II window, much higher than that measured in NIR-I window, 2.94 (Figure 4E and F). Statistical analyses also revealed a significantly higher SBR (Figure S2C, P < 0.001) and lower FWHM (Figure S2D, P < 0.001) in the NIR-II window than those in the NIR-I window.

### NIR-II imaging for normal femoral vessel system

**Figure 5:**
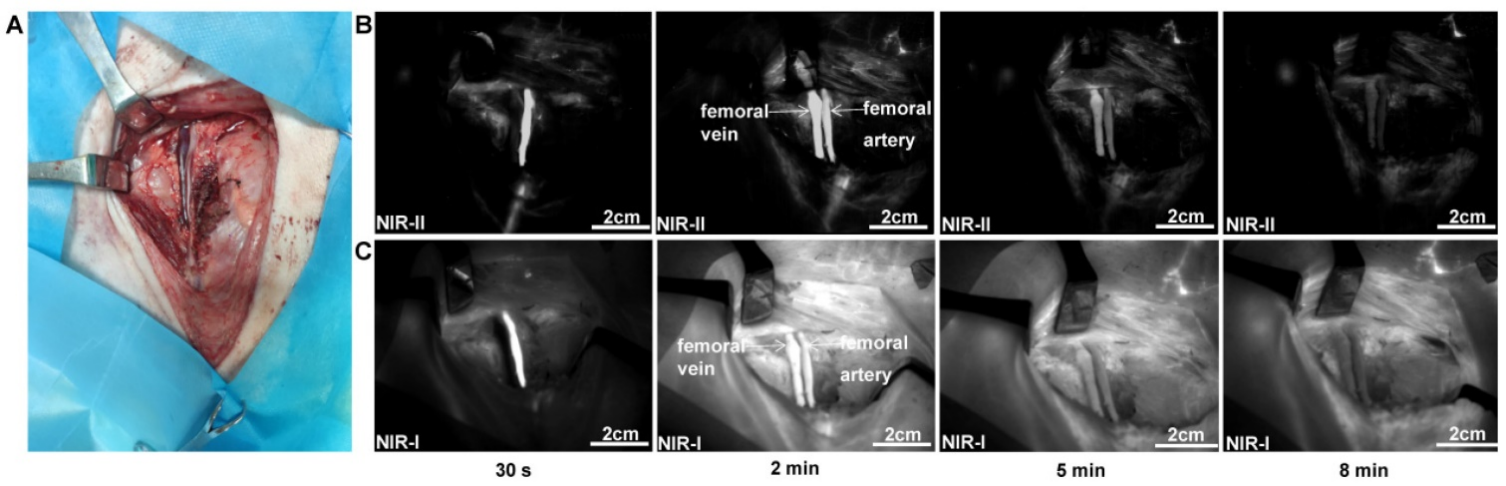
** NIR Imaging for normal femoral vessel system. (A)** Bright-field image of normal femoral vessels anatomy of the pig. **(B)** Continuous dynamic fluorescence imaging in the NIR-II window from 30s to 8 minutes after intravenous injection of ICG. **(C)** Corresponding NIR-I window imaging.

The femoral artery and femoral vein were exposed (Figure 5A), and quickly identified under the NIR-II detector after intravenous injection of ICG (Figure 5B). The time course of NIR-II angiography could be divided into arterial and venous phases. The arterial fluorescence appeared firstly, peaked at about 30 seconds after injection, and then became quite indistinguishable about 8 minutes after injection. In contrast, venous fluorescence appeared slowly and persisted for a longer time. Movie S3 shows this in more detail. Compared to NIR-II imaging, NIR-I imaging exhibited higher background fluorescence (Figure 5C).

### NIR-II imaging for the identification of femoral venous thrombus

**Figure 6:**
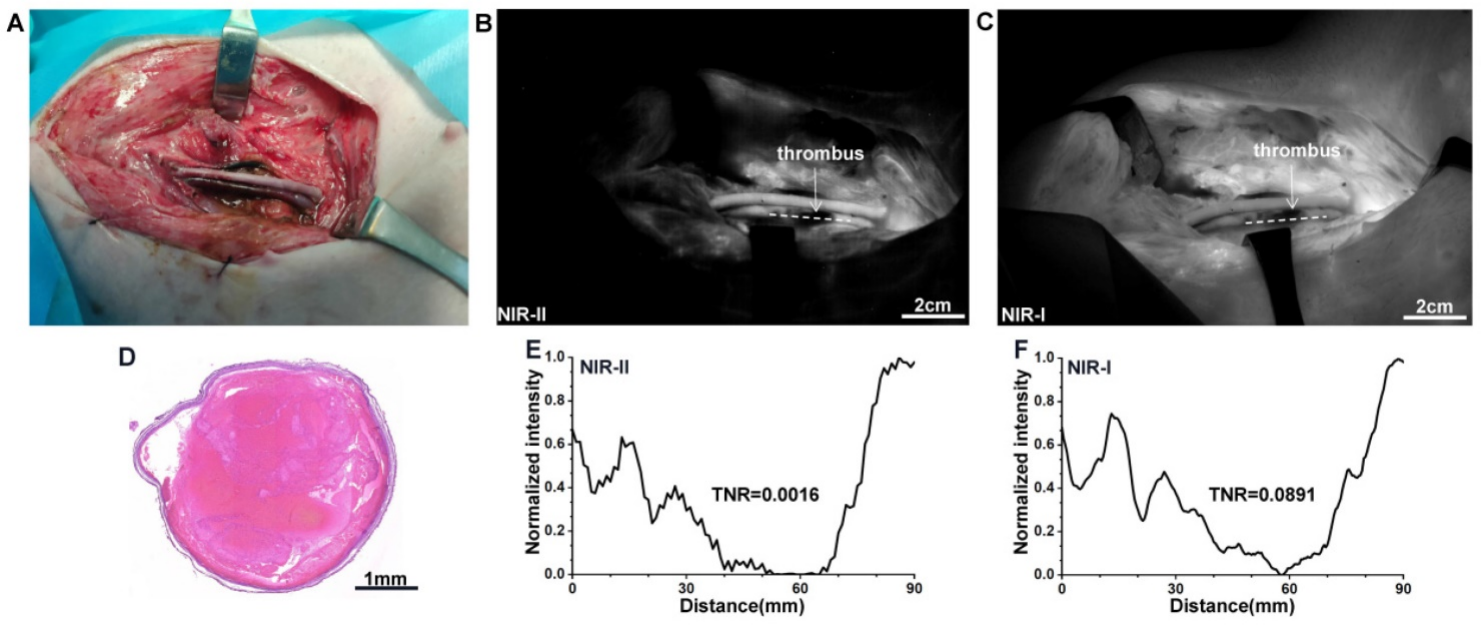
** NIR Imaging for the identification of femoral venous thrombus. (A)** Bright-field image of femoral venous injury by FeCl3. **(B and C)** Fluorescence images of venous thrombus in NIR-II and NIR-I windows, respectively. **(D)** The venous thrombus was further confirmed by H&E stain. **(E and F)** The TNRs of the thrombus (white dash lines in Figure **B** and **C**) were calculated, respectively.

The injury in pig's femoral venous was established to induce the thrombus formation (Figure 6A). Under NIR imaging, the thrombus appears as a coloboma-like signal (Figure 6B and C). The thrombus site was also confirmed by H&E stain (Figure 6D). The TNR (thrombus-to-normal venous ratio) of the thrombus marked by a white dash line obtained from NIR-II imaging was 0.0016 (Figure 6E), much lower than that obtained from NIR-I imaging, 0.0891 (Figure 6F). Statistical analyses revealed a significantly lower TNR in the NIR-II window than that in the NIR-I window, P < 0.001 (Figure S2E).

### NIR-II imaging for the detection of vascular patency during anastomosis

**Figure 7:**
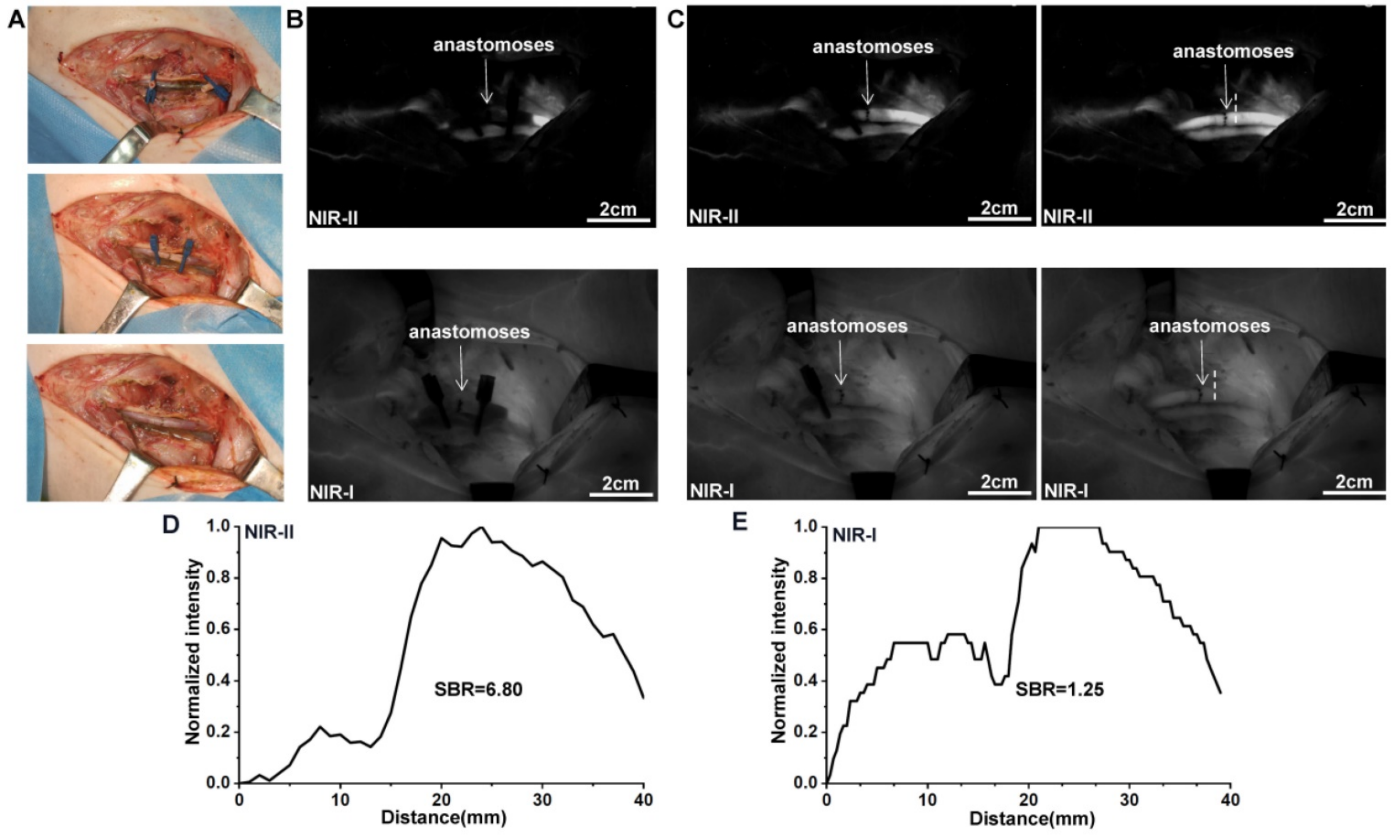
**NIR Imaging for the assessment of vascular patency during anastomosis. (A)** Intraoperative real-time views of vessel anastomoses. **(B-C)** NIR-II window and NIR-I window images for the detection of pig' s femoral artery patency. The blood quickly passed through the anastomoses after removing the vascular clamps. **(D and E)** The SBRs of anastomoses (white dash lines in Figure **C**) in NIR-II and NIR-I imaging were calculated, respectively.

The swine model for anastomosis with femoral artery rupture was established in Figure 7A. As ICG was injected intravenously after anastomosis, blood flow within the proximal and distal parts of the femoral artery was rapidly visualized by NIR-II window imaging (Figure 7B). The blood flow quickly passed through the anastomoses while removing the vascular clamps on both sides of the broken end (Figure 7C). Movie S4 shows this in more detail. The anastomoses marked by a white dash line visualized in NIR-II window also demonstrated an increased SBR (defined as the anastomoses-signal-to-adjacent tissue ratio) compared to NIR-I window (6.80 vs 1.25; Figure 7D and E). Statistical analysis also revealed a significantly higher SBR in the NIR-II window than that in the NIR-I window, P < 0.001 (Figure S2F). The results of NIR angiography also matched with the vascular patency test.

## Discussion

Fluorescence imaging in NIR-II window, with deeper penetration, higher spatial resolution and imaging contrast, has been recognized as a superior alternative to the well-established NIR-I fluorescence imaging 5, 6. In this preclinical study, we constructed a portable NIR-II imaging system and achieved clearer visualization for the lymphatic and vascular systems of pigs dynamically in NIR-II imaging than that in NIR-I imaging, including lymphatic drainage, lymphatic obstruction, lymphorrhea, and venous thrombus formation, by using the tail fluorescence of ICG. In addition, we firstly used ICG-NIR-II imaging to detect the patency of vascular anastomoses and lymphatic reconstruction after flap transplantation, providing a basis for the application of this approach in microsurgery and reconstructive surgery.

Supermicrosurgical lymphatic anastomosis and lymphaticovenular anastomosis are important surgical methods for preventing and treating secondary limb lymphedema and lymphorrhea 37, 38. It is crucial to locate the leaking site or find appropriate lymph vessels for anastomosis before surgery. However, lymph vessels are usually small and easily confused with surrounding tissues, making them hardly identified with naked eyes. Regional lymph node involvement is an essential prognostic index in cancer patients 39-40. Accurate SLNs identification and dissection are the standard treatment for current cancer management and have been widely used in tumor surgery 41. The existing standard method for lymphoscintigraphy and SLN localization is a dual technique involving the injection of a technetium-99m (99mTc)-labeled nanocolloid and blue dye 42. Whereas, radioactive colloid exhibits certain disadvantages, such as the need for specialized nuclear doctors, high costs, and the lack of visual information. In addition, the blue dye staining can only determine lymph vessels and SLNs after incising the skin during operation 43, and cannot provide information to doctors before operation. In our study, ICG-NIR-II imaging could provide real-time visualization of lymphatic drainage and necessary information for preoperative surgical planning without radiation, especially for the precise choice of surgical incision. More importantly, preoperative imaging for lymphatic drainage demonstrated that NIR-II imaging shows better image quality than NIR-I imaging, with lower FWHM and higher SBR. Furthermore, combined with methylene blue staining, lymphatic vessels and lymph nodes could be quickly and accurately detected during operation. By combining ICG-NIR-II imaging and blue dye staining, this new dual tracing modality may exhibit great potential as an alternative to traditional standard mapping methods and can minimize inaccurate incision and dissection as much as possible.

Lymphatic drainage disorder is considered as the primary mechanism of postoperative edema of flaps 44. However, there is still a lack of research on lymphatic drainage after flap transplantation. Therefore, the non-invasive display of the lymphatic reconstruction inside the flap is vital to clarify the nature of the flap edema, and it can also provide a theoretical basis for the treatment. As the flap became edematous early after surgery, the skin and subcutaneous tissue appeared thickened. In this deeper penetration needed condition, NIR-II imaging of regenerative lymph vessels inside the flap was clearer than NIR-I imaging, due to the deep tissue penetration capability. These results were also consistent with the quantitative analysis of FWHM and SBR. And even more interestingly, we found the lymphatic regeneration within the flap of pigs as early as 7 days postoperatively. To our best knowledge, this is the first report of dynamically monitoring lymphatic regeneration in flaps by NIR-II imaging, enriching the basic research of flaps. In addition, the edema the flap would be gradually reduced with the increase of regenerated lymph vessels. However, due to the limited samples, we do not know the maximum regeneration capacity of lymphatic vessels in the flap. Future researches are warranted to shed more light on this phenomenon and investigate its mechanism.

Providing real-time imaging of thrombus formation with high spatial and temporal resolution is significant during surgical thrombectomy. Nevertheless, current methods for imaging vascular structures such as CT and MRI are limited by long scanning and post-analyzing times and poor temporal resolution 5, 6. More importantly, these are not convenient for real-time observation during operation. Although Doppler sonography can be used intraoperatively, its poor spatial resolution hinders its application 36. In our study, we demonstrated that the standard vascular structure and venous thrombosis could be distinctly visualized in real-time by ICG-NIR-II imaging during operation. In addition, the TNR of NIR-II imaging was much lower than that of NIR-I imaging, indicating that NIR-II imaging discriminated thrombus more effectively than NIR-I imaging and may be a promising diagnosis for future intraoperative thrombus localization in clinic. Furthermore, this approach showed excellent potential to help us explore other vascular malfunction, especially if one vessel has multiple branches, and could avoid the risk of bleeding associated with excessive dissection.

One of the critical issues in vascular anastomosis is the intraoperative assessment of vessel patency. Unfortunately, a direct intraoperative inspection may fail to reliably detect vessel patency, because it is not known whether the distal blood flow is provided by the proximal blood flow or by other collateral circulation by naked eyes. The patency test is a common way to evaluate vascular patency during anastomosis, but there is a risk of damaging the intima 45. Doppler sonography requires contact with blood vessels and may also cause damage to the anastomoses. Previous studies have shown that ICG-NIR-I imaging can help surgeons detect anastomotic failure 19, 46. In our study, ICG-NIR-II imaging outperformed the ICG-NIR-I imaging in patency detection, with higher SBR. Moreover, a little ICG would somewhat remain in the intima of vessels after repeated ICG injection, resulting in a permanent intraluminal fluorescence in NIR-I imaging, while almost no fluorescence in NIR-II imaging (Figure 7B). It is mainly attributed to the peak fluorescence emission intensity displayed by ICG in the NIR-I window, rather than its tail emission in the NIR-II window. However, the higher sensitivity for the residual ICG in NIR-I imaging will interfere with the following examination of the anastomotic patency. Therefore, these results demonstrated that NIR-II imaging was superior to NIR-I imaging in assessing anastomoses patency and could possibly improve the quality of anastomosis.

## Conclusion

The current work highlighted the first application of a portable NIR-II imaging system for dynamically monitoring both lymphatic and vascular systems in physiological and pathological conditions using a swine model which more closely represents humans in size and complexity. Under different simulated clinical scenarios, our portable NIR-II imaging system was simple to manipulate and showed excellent reliability. These data suggest that ICG-NIR-II imaging exhibits great prospects for clinical translation in the diagnosis of malfunctions in lymphatic and vascular systems and the surgical navigation of microsurgery and reconstructive surgery without NIR-II fluorophores approved by the FDA, and better imaging quality by this approach could be also achieved than ICG-NIR-I imaging.

## Supplementary Material

Supplementary figures and movie legends.

Supplementary movie 1.

Supplementary movie 2.

Supplementary movie 3.

Supplementary movie 4.

## Abbreviations

CT: computed tomography
EPR: enhanced permeability and retention
FDA: Food and Drug Administration
FWHM: full-width-half-maximum
H&E: hematoxylin-eosin
ICG: indocyanine green
InGaAs: indium gallium arsenide
MRI: magnetic resonance imaging
NIR: near infrared
SBR: signal to background ratio
SLNs: sentinel lymph nodes
TNR: thrombus-to-normal venous ratio
US: ultrasonography
