# Supplementary material for: Dynamically monitoring lymphatic and vascular systems in physiological and pathological conditions of a swine model via a portable NIR-II imaging system with ICG
Source: Int J Med Sci. 2022 Oct 17;19(13):1864–74. doi: 10.7150/ijms.71956 (PMC9682514; doi:10.7150/ijms.71956)
Supplement: Supplementary file 1 — Supplementary figures and movie legends. [file ijmsv19p1864s1.pdf]

Supplementary figures

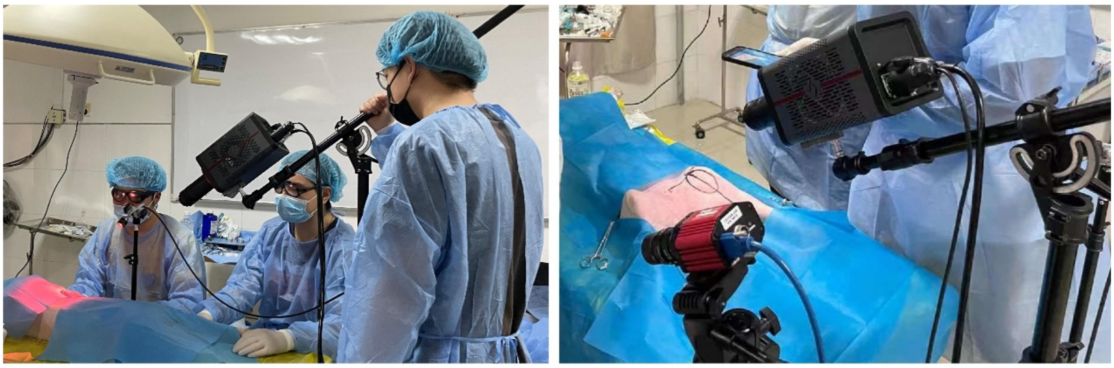

Figure S1. The portable NIR-II imaging system in operation.

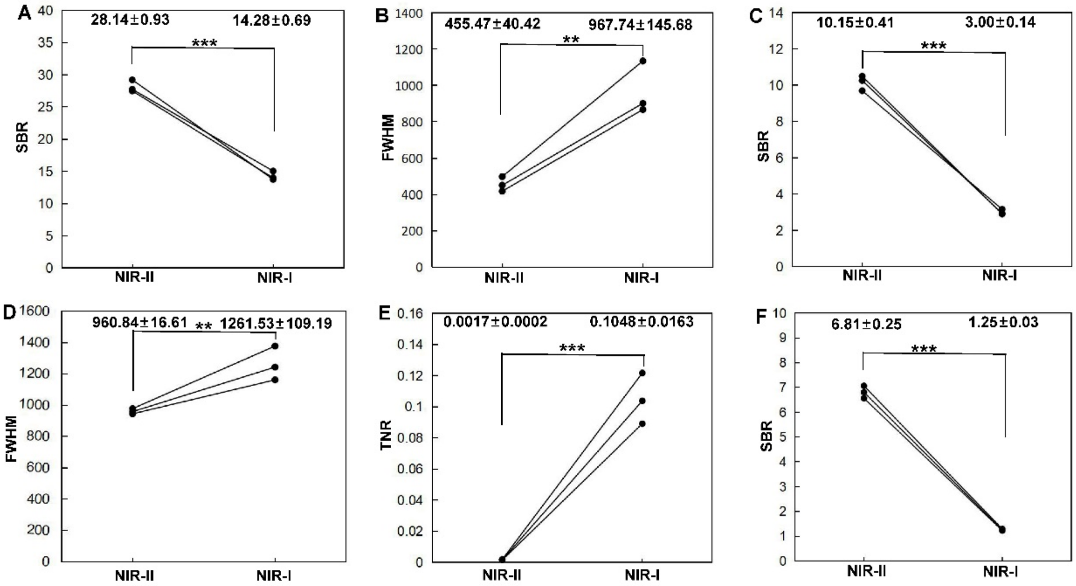

Figure S2. Comparison of SBR/TNR/FWHM for NIR-I and NIR-II imaging in different conditions. (**A, B**) Quantitative analysis of the SBR and FWHM of NIR-I and NIR-II sub-surface lymphatic vessels imaging in normal models (n = 3). (**C, D**) Quantitative analysis of the SBR and FWHM of NIR-I and NIR-II sub-surface lymphatic vessels imaging in saphenous artery flap models (n = 3). (**E**) Quantitative analysis of the TNR of NIR-I and NIR-II thrombus imaging in femoral venous thrombus models (n = 3). (**F**) Quantitative analysis of the SBR of NIR-I and NIR-II anastomoses imaging in femoral artery rupture models (n =3).

Supplementary movie legends

Movie S1 Dynamic NIR-II imaging for preoperative mapping of lymphatic channels and sentinel

lymph nodes with a 1100-nm long-pass filter in a swine model.

Movie S2 Dynamic NIR-II imaging for monitoring lymphatic reconstruction in the saphenous artery flap with a 1100-nm long-pass filter in a swine model.

Movie S3 Dynamic NIR-II imaging for femoral vessels system with a 1100-nm long-pass filter in a swine model.

Movie S4 Dynamic NIR-II imaging for the detection of vascular patency during anastomosis with a 1100-nm long-pass filter in a swine model.
